# A qualitative study on mixed experiences of discrimination and healthcare access among HIV-positive immigrants in Spain

**DOI:** 10.1186/s12889-021-10388-6

**Published:** 2021-02-19

**Authors:** Megi Gogishvili, Karen R. Flórez, Sergio A. Costa, Terry T.-K. Huang

**Affiliations:** Center for Systems and Community Design, Graduate School of Public Health and Health Policy, City University of New York, 55 West 125 Street, New York, NY 10027 USA

**Keywords:** HIV-positive immigrants, Systematic barriers, 2012 RDL and RD, Access to care, Immigrants, Positive impact of third-party guidance on facilitating access to care

## Abstract

**Background:**

Immigrants are disproportionally impacted by HIV infection in Europe and in Spain. Immigrants are also identified as a vulnerable population during economic crises. Various socioeconomic barriers hinder HIV-positive immigrants from accessing healthcare services in the host country. As a result of the 2008 financial crisis, Spain has implemented multiple austerity measures, one of which was the enactments of Royal Decree Law (RDL) 16/2012 and Royal Decree (RD) 1192/2012 which abolished universal healthcare coverage. In this context, this study examined: 1) Participants’ mixed experiences in accessing health care after the enactment of 2012 RDL and RD, and 2) Distress felt by the participants and their experiences as HIV-positive immigrants living in Spain.

**Methods:**

Participants were recruited through a nongovernmental organization (NGO) during routine visits at the center. A total of 12 participants were interviewed to reach data saturation. Participants were HIV-positive immigrants living in Spain for 1 or more years, allowing for substantial experience with navigating the healthcare system. Thematic analysis was performed to identify common themes in participants’ experiences living as HIV-positive individuals in Spain and in accessing healthcare.

**Results:**

Four primary themes were identified. The primary systemic barrier to accessing health care encountered by participants was the inability to fulfill the requirement of having proof of registration in an Autonomous Community for the required time period, thus not being able to apply for a public health insurance card and utilize free care services. Participants identified a positive impact of third party (NGO, social worker, friend/family member) guidance on their experience of applying for a public health insurance card. Participants expressed experiencing emotional or physical (eg, side effects of medication) distress in adapting to life as HIV-positive individuals. Participants also identified experiencing discrimination while living as HIV-positive immigrants in Spain.

**Conclusions:**

HIV-positive immigrants are underserved in Spain. They encounter systemic barriers while accessing healthcare services, and experience fear and/or discrimination. The study underscores the role of NGOs in helping HIV-positive immigrants navigate the healthcare system. More research is needed on comprehensive approaches to address healthcare needs of HIV-positive immigrants in Spain.

## Background

More than half of European Union (EU) member states identify immigrant populations as disproportionately affected by HIV [1–4]. Since the mid-1990s, Spain has reported a disproportionate number of HIV cases among immigrants as compared with native-born Spaniards [1–6]. According to the first nationwide HIV data collection tool (Information System for New Diagnosis of HIV) implemented in Spain, of 3366 newly diagnosed cases (7.2 cases per 100,000) in 2014, 32% (1077 cases) were identified among immigrants [7]. However, the prevalence of HIV among immigrants had been low historically [1, 8]. The recent increase in HIV prevalence among immigrants has been attributed to the drastic growth of migration in the last decades [9, 10]. In 1998, immigrants comprised only 1.6% of the total population, whereas in 2019 that percentage increased to 10.7% [11]. In 2019, immigrants from the EU comprised 36.3% of the total immigrant population in Spain (5,025,265), followed by Africa (22.3%), South America (19%), Asia (9.3%) and Central America and the Caribbean (5.9%) [11].

Among all immigrant population in Spain, Latin American and Sub-Saharan immigrants have the highest number of newly diagnosed HIV cases [4, 6, 7, 10]. These two groups have also the highest HIV incidence among all Western European countries [4]. Men who have sex with men (MSM) have the highest transmission rate among Latin American immigrants, whereas heterosexual transmission is the primary mode among Sub-Saharan Africans [6–8]. Sub-Saharan Africans and Latin American immigrants were also disproportionally represented among cases of late diagnosis in twelve ACs of Spain between 2003 and 2008 [12]. Almost 50% of immigrants diagnosed with HIV in Spain were already in need of ART treatment (pointing to late diagnosis) compared to approximately 40% of the native-born population [2]. Nevertheless, only 4% of all HIV-positive immigrants were receiving ART in 2001, 10% in 2008, and 14% in 2010 [2]. Similar percentages of delayed diagnoses among immigrants and native-born were reported in 2014 [7]. It is evident that necessary treatment is not reaching or is delayed for immigrants, compared to the native-born population in Spain.

Socioeconomic insecurity, legal status of immigrants, and the experience of stigmatization in the host country affect immigrants’ access to prevention programs, testing, and care services [2, 13]. This further creates inequalities in healthcare access among immigrants relative to the native-born population [14], and increases their vulnerability to HIV infection [4, 5, 13–18]. Immigrants were also found to engage in high HIV-related risk behaviors such as high alcohol consumption, inconsistent condom use, drug injection [19–23], and sexual exploitation employment [24] due to the social and economic difficulties they face in their host country. Due to the encountered difficulties, immigrants tend to prioritize food and housing over health [13], further impeding the possibility of early HIV detection or successful treatment. Other important barriers that increase the vulnerability of immigrants to HIV include restrictive laws and regulations that deny or limit immigrants’ right to health [4, 13, 25, 26]. Overall, health policies [27] and economic crises [28–30] can have a significant effect on population health. The impact of economic crises on vulnerable groups, such as immigrants, is disproportionally severe [31].

In recent years, Spain has executed multiple austerity measures to cope with the effect of the 2008 economic crisis. In 2012 the government enacted Royal Decree Law (RDL) 16/2012 and Royal Decree (RD) 1192/2012 that, together with other budget cuts, increased copayments for an already economically distressed population, denied the right to health care among the undocumented immigrant population [31–33], and altered the previously exercised universal healthcare system in Spain [31–34]. Specifically, it has been estimated that approximately 500,000 undocumented immigrants were excluded from the national health system of Spain as a result of the 2012 health reform [33]. These undocumented immigrants were left with the option of purchasing health insurance. However, the premiums of health insurance are generally unaffordable for undocumented immigrants [30], considering that 40% of documented immigrants in Spain are unemployed [35]. Thus, the new law created more barriers for immigrants while accessing necessary healthcare services and potentially added to discrimination or stigma commonly experienced due to HIV-positive status in a host country [2].

As a response to the 2012 health reform, some autonomous communities (including Valencia), due the decentralized nature of the Spanish health system, developed various regional regulations and instructions to still provide access to free healthcare to immigrants (Gogishvili et al., unpublished data). In December 2013, the Ministry of Health, Social Services and Equality also published new regulatory change under Royal Decree 576/2013 that granted all individuals (including undocumented immigrants) access to free healthcare in cases of diseases subject to epidemiologic control (including HIV/AIDS) [36]. The inconsistencies and frequents changes in relevant laws and how they were implemented in each region likely created confusion among patients and health professionals.

In this context, and in light of both the social disadvantage of and the paucity of data on HIV-positive immigrants in Spain, this pilot qualitative research aimed to explore: 1) the mix of experiences encountered by HIV-positive immigrants in Spain while accessing health care after the enactment of 2012 RDL and RD, and 2) any distress or discrimination experienced by participants as HIV-positive immigrants living in Spain.

## Methods

### Participant recruitment

Twelve participants were recruited by a local nongovernmental organization (NGO) through phone or in-person contact during their routine visits to the office. The local NGO is dedicated to helping HIV-positive immigrants (e.g., providing psychologic support as well as necessary nutritional food) and to guiding them through the process of accessing healthcare services in Valencia, Spain. To ensure participant anonymity, the name of the NGO is not listed. A purposive on-going sampling was performed by a social worker employed at the local NGO, otherwise not involved in the research, who identified HIV-positive immigrants that routinely engaged with the organization and met the criteria determined by the study. The social worker explained the purpose of the study (the two objectives) to prospective participants and informed them that the research was conducted by a doctoral student of the City University of New York Graduate School of Public Health and Health Policy. Refusal to participate in the study did not impact services provided by the NGO. Informed consent was obtained. Participants were included in the study if they met the following criteria: 1) adults 18 years or older; 2) HIV-positive; 3) had been living in Spain for 1 or more years; and 4) had experience accessing necessary healthcare as HIV-positive adults. The recruitment process was stopped when data reached saturation. All interviewees were given €10 to participate in the research. The effect of a monetary incentive was discussed during the design of the study; however, the small amount was considered appropriate compensation for the participants’ time and effort and was unlikely to be coercive. The study was approved by the City University of New York Institutional Review Board (IRB).

### Interview procedure

Semi-structured interviews were conducted at the partner NGO site in summer 2019 to facilitate in-depth conversations with study participants around sensitive topics. This method allows for a deeper understanding of participants’ life experiences [37].

Interviews were conducted in-person and in Spanish by the lead author, then a doctoral student at the City University of New York Graduate School of Public Health and Health Policy. The interviewer also held a master’s degree in social work. Both degrees equipped her with skills to perform high-quality interviews with the utmost sensitivity to the topic and to participants. Participants had no prior encounter with the interviewer and no one else was present during the interview process. Participants agreed to the recording of the interview to ensure the accuracy and completeness of the data captured. Thus, there was no need to carry out repeat interviews or send out transcripts of the interviews for confirmation. The findings of the research were also not sent to the participants for feedback, to maintain authenticity of natural ‘story telling’ and avoid the influence of the participants possibly overthinking their answers after reading the results. To not distract the interviewees and to demonstrate respect to them by the researcher, notes about participants or the process were made only after the interview.

Semi-structured interviews were designed to explore participants’ experiences in the following 2 areas: 1) life as an HIV-positive person in terms of emotional or physical distress, and perceptions or experiences of discrimination; and 2) experiences in accessing healthcare services in Spain, and how/if they were able to overcome them. The semi-structured interview guide (see Appendix 1) was designed in accordance with a conceptual framework by Lévesque et al. [38] that concentrated on different levels of healthcare access, including participants’ ability to identify healthcare needs, seek care, reach necessary services, obtain care, and receive adequate services. The Lévesque et al. [38] framework has been previously validated among immigrants in various European countries [39]. Our interview guide also included questions exploring participant experiences when diagnosed as HIV-positive and the social effect it had on them. The final question in the guide addressed participants’ knowledge of 2012 RDL and RD and if/how these impacted them. As this study was pilot in nature, no pre-piloting of the interview guide was conducted. That said, the interview guide used was similar to that of prior research among HIV-positive immigrants in various European countries [39].

### Thematic analysis

Before performing analysis, all interviews were transcribed and de-identified. Transcription of the interviews was done by a professional organization that specialized in transcribing audio material into text in Spain. Both transcription and subsequent analysis were performed in Spanish. Only quotes used in this paper were translated by the lead author from Spanish into English. Quotes were corrected for grammar and restructured for clarity without altering the meaning of the original speech. After in-depth review of the transcripts and audio of the interviews, data were coded by the lead author (see Coding Tree in Appendix 2).

Thematic analysis identified commonalties among the participants. Thematic analysis was chosen for this study as it allowed for capturing the richness of stories [40], as opposed to using a predetermined framework to analyze the data. The interviews were analyzed following Braun and Clarke’s [40] guidelines for thematic analysis. Dedoose (version 8.3.16) was used to aid the analysis.

## Results

### Characteristics of the participants

Data were considered sufficiently saturated after in-depth interviews with the 12 study participants. Another two participants dropped out of the study at the last minute due to a change of mind in their interest in the topic. The interviews lasted 25–45 min. Table 1 shows the characteristics of the participants. Participants were from 6 different countries (Argentina, Chile, Cuba, Honduras, Peru, and Venezuela) and all spoke Spanish as a native language. Five of the 12 participants were employed and 4 were married or in a relationship. Sex distribution was approximately equal among women and men; the majority of the participants were between the ages of 31 and 50 years. Five participants were heterosexual, 5 were homosexual, and 2 identified no sexual preference. All participants were diagnosed HIV-positive in their home country. Half of the participants had lived with HIV from 6 to 25 years. Half of the participants had resided in Spain from 1 to 5 years and the rest for 6 or more years. Regarding immigration status, more than a half of the participants arrived in Spain without documentation and the majority of participants were documented at the time of the interview. Half of the participants arrived for the first time in Spain alone. Reasons for immigration varied among the participants; most reasons related to financial or political hardship.

**Table 1:** Characteristics of the Participants

**Sex**	
Women	6
Men	5
Transgender women	1
**Age, y**	
18–30	2
31–50	7
51–65	3
**Sexual orientation**	
Heterosexual	5
Homosexual	5
Unknown/other	2
**Region of origin**	
Argentina	2
Chile	1
Cuba	3
Honduras	1
Peru	1
Venezuela	4
**Employment status**	
Employed	5
Unemployed	2
Part-time employment	1
Illegal manual work	3
Retired	1
**Family status**	
Single	8
Married	2
In a relationship	2
**Number of years diagnosed with HIV**	
< 5	4
6–15	1
16–25	5
26>	2
**Probable transmission mode**	
Heterosexual contact	5
Homosexual contact	5
Unknown	2
**Initially tested in Spain**	
Yes	1
No	11

Table 2 shows the participants’ brief immigration journeys. Table 3 summarizes participant experiences with being HIV-positive in Spain and in their countries of origin.

**Table 2:** Immigration Details of the Participants

**Time living in Spain, y**	
1–5	6
6–11	1
12–16	2
17>	2
Unclear	1
**Documentation status when arrived**	
Documented	5
Undocumented	5
Asylum seeker	2
**Documentation status now**	
Documented	7
Undocumented	3
Asylum seeker	2
**Immigrated to Spain alone**	
Yes	6
No	6
**Living with partner/family member**	
Yes	6
No	5
Unknown	1
**Reason(s) of immigration**	
Financial situation of country of origin	3
Political situation of country of origin	3
Health reasons	1
Political situation and health reasons	1
Other (Spanish partner, threat to life in the country of origin, financially supporting child already living in Spain, seeking treatment for a family member)	4

**Table 3:** Participants’ Experiences Being HIV-Positive

**Family/friend support on the matter of HIV**	
Full support (family and friends)	2
Family support	1
Friend support	2
Some support (some family and/or some friends)	6
No support	1
**Experience/perception of country of origin context for HIV**	
Lack of medication	5
Discrimination (including fear of discrimination)	2
Stigma and prejudice (including lack of information)	4
No experience in country of origin	1
**Received initial guidance on how to access necessary healthcare services in Spain**	
NGO guidance (including associations)	6
Hospital staff (social worker)	1
Friend guidance (friend, family member)	5
**Experience of applying for insurance card in Spain**	
Positive	9
Negative	3
**Perceived reasons for negative experience while trying to access healthcare services or after receiving care**	
Hospital staff (not entitled to health insurance card)	1
Not entitled by the law (3-month registration requirement not met)	2
Not explained the system in advance (received a bill)	1
N/A (participants who did not identify negative experience)	8
**Perceived experience while trying to access other health services/follow-ups**	
Positive	9
Negative	1
Unknown or not applicable	2
**Emotional experience of living with HIV**	
Distress	4
Distress in the initial stages	5
No negative thoughts expressed	3
**Experience of discrimination in country of origin**	
Fear of discrimination	5
Experienced discrimination	2
No experience of discrimination	1
Other (unknown or not applicable)	4
**Experience of discrimination in Spain**	
Fear of discrimination	2
Experienced discrimination	2
No experience of discrimination	3
Other (felt differential treatment, unknown, unclear)	5
**Knowledge/experience of 2012 RDL and RD**	
Aware and did not change anything	1
Somewhat aware and did not change anything	3
Not aware and was not in the country	4
Not aware and was in the country	1
Not aware and unclear if he/she was in the country	3

*NGO* nongovernmental organization, *RD* Royal Decree, *RDL* Royal Decree Law

### Main themes identified

Table 4 presents 4 themes identified during the interviews. All 4 themes address life experiences of the participants and the systemic barriers and/or enablers they encountered while initially trying to access healthcare services in Spain.

**Table 4:** Themes Identified through the Interviews

Theme	Brief Description
Barriers encountered when initially trying to access free healthcare services in Spain	Participants experienced systematic barriers (not being eligible to receive public health insurance card) and administrative barriers (front office staff at the hospitals) while trying to access free healthcare services in Spain.
Possible reasons of positive experience when initially trying to access free healthcare services in Spain	Participants identified sources who gave them initial guidance on the process of receiving free healthcare services and were perceived by the interviewer as a possible reason of overall positive experience. Specifically, guidance from an NGO, from a friend, hospital staff, or personal effort.
Experienced distress after being diagnosed HIV positive	Participants experienced emotional or physical distress and/or struggled to adapt to living as an HIV-positive person during initial period of diagnosis, throughout life, or described no negative time in their lives as it pertains to the adjustment to being HIV-positive.
Perceived and experienced discrimination due to being HIV-positive while in Spain	Participants had perceived fear of discrimination, experienced discrimination, experienced differential treatment, ignorance about HIV, as well as described not having any discriminative incidents as an HIV-positive person.

*NGO* nongovernmental organization

#### Theme 1: barriers encountered when initially trying to access free healthcare services in Spain

A minority of participants had negative or somewhat negative experiences while trying to access free healthcare services. Not being able to receive a public health insurance card was noted as a specific factor. Participant 3 discussed problems with meeting the requirement of being registered in an Autonomous Community (AC) for the minimum months required in order to apply for a public health insurance card.

Participant 3. *“Applying for a public health insurance card was a problem at the start, because I arrived in Spain and here it is fundamentally important to be registered in an AC. I spent 4 months without meeting someone who would register me in the AC. I was paying rent but no one ever registered me there. I got registered in my fourth month in the AC.”*

Similarly, Participant 11 described problems with meeting the requirement of being registered in an AC in order to apply for a public health insurance card.

Participant 11: *“Everything had to be done one after another. [Blood] analysis, applying for a public health insurance card especially, because I was not previously registered 3 months in the AC as they required. I did not have that yet. They asked me for the proof of registration in the AC and I just gave an address of the place I arrived to.”*

Another participant had difficulty getting an appointment with the social worker at the hospital to apply for the public health insurance card. According to the participant, he thought the social worker could grant a public health insurance card, as advised by a friend.

Participant 6: *“The admission and information desks at the hospitals need a reason why you want an appointment with the social worker. They were telling us different reasons why they could not give us an appointment with the social worker. They told us that in order to get such an appointment we already needed to have a public health insurance card. We explained that we did not have the card and that is why we wanted an appointment, but they told us that the social worker was not for that. We went there because our friend received the card this way, but in another institution or health center.”*

When the interviewee was asked if the admission or information staff at the hospital explained where they should have gone to apply for the card, Participant 6 answered *“No, they never told us.”*

The majority of participants expressed limited to no barriers while trying to access healthcare services in Spain. For example, Participant 2 shared the following:

Participant 2: *“( …*) *I was talking to the people with the truth, I was telling them, “listen, we are trying to have a normal life in Spain, have all of our documents in order, we do not want anyone to give us gifts”, but it could have been because I am HIV-positive they were immediately giving me an pubic health insurance card with validity of 3 months, 6 months, depending on the regional government (…*).”

Participant 6 also expressed experiencing no barrier in receiving a public insurance card necessary to access free healthcare services in Spain.

Participant 6: *“Here in Spain, I was given one paper that allows me to live in Spain so I did not have any problems, look here it is ( …) for all the hospitals.”*

Overall, only 1 participant was aware of the 2012 reform that restricted free access to healthcare services among immigrants in Spain, though this participant did not experience any barrier when accessing healthcare. Three participants were somewhat aware of the 2012 healthcare reform but thought that these changes did not impact their lives. Half of the participants (4 of 8) who were not aware of the healthcare reform had not yet immigrated to Spain when the 2012 Royal Decree-Law and Royal Decree were implemented.

#### Theme 2: possible reasons of positive experience when initially trying to access free healthcare services in Spain

This theme expresses the notion of a “guiding source” who possibly influenced participants’ positive experiences when initially trying to navigate the public health system in Spain. The majority of the participants mentioned guidance from a local NGO who showed them pathways to receiving free healthcare services and a public health insurance card. They found an NGO through personal effort or a friend. Participant 11 described her experience of trying to receive treatment for HIV in Spain through the help of a local NGO, as follows:

Participant 11: *“I came here with enough medication for one month. When it was gone, I thought, “What do I do now?” I searched on the Internet and I said, “There should be some organization here dedicated to HIV-positive people that can help me in this situation.” I found AVACOS-H. I called them and they answered and asked me, “Can you come in now?” Of course, I went because I had been without medication for 2 weeks. When I explained my situation and how I was, Diana helped me a lot. She immediately talked with a doctor for me.” (AVACOS-H is a Valencian Association dedicated to HIV, AIDS, and hepatitis.)  *

Similarly, Participant 10 described how everything was organized for him by a local NGO as follows:

Participant 10: *“The Red Cross guided me to receive a public health insurance card: right away they helped me talk with a doctor who gave a referral, after which I went to ambulatory care and talked with a social worker to whom I explained my health needs and that I needed treatment. Right away I was given a health insurance card and a doctor gave me the treatment I needed.”*

Several participants shared how friends guided them through the process and helped navigate the system. For example, Participant 1 expresses how a friend, who immigrated to Spain before him, explained to him where to go and what to do.

Participant 1: *“I came here with help from my friend from Cuba. He came here two months before I did. Thus, he already navigated all these formalities and he explained to me what I had to do. I went to the medical center. But I was not registered in the AC. I explained that I was HIV-positive and that I had to take a medication. I talked with a social worker and she processed everything for me.”*

In the same vein, Participant 12 noted the following:

Participant 12: *“A man that I was married to was coming to Valencia ( …) When he arrived, he told me “do not pay anything.” I received a bill at the hotel where I was staying. He told me, “Do not pay anything and this will be resolved.” I went back with him [to the hospital] and that is when I started doing all the paperwork and public health insurance card...”*

Similarly, Participant 9 talked about how a friend helped her complete the requirements to receive a public health insurance card and guidance received from a local NGO to pursue other possible help.

Participant 9: *“Yes, the woman in the apartment where I was living helped me get proof of registration at this address. AVACOS-H also helped to see if I could receive financial assistance.”*

All participants shared having some level of guidance from a third party (such as an NGO, hospital staff, or a friend) that helped them navigate the Spanish healthcare system.

#### Theme 3: experience of distress after being diagnosed HIV-positive

The majority of participants expressed difficulty with accepting becoming HIV-positive, with managing the health effects of having HIV or of its treatment, and with sharing the diagnosis with loved ones. For example, Participant 2 shared the following anecdote:

Participant 2: *“At the start I was very depressed. In my case I had a shock and it took me 15 years to talk about it, and I talked about it in AVACOS-H. It was incredible horror, panic, the world came down on me, I had adolescent children, and they were in the waiting room at the hospital. One of them heard the diagnosis and told the other. They found out like this, because if it was for me, I would just have carried this alone.”*

Similarly, Participant 4 described experiencing distress due to the process of accepting the diagnosis and sharing it with loved ones.

Participant 4: *“My experience when I came here was very bad, emotionally. This man, at the NGO, who I think is a psychologist, talked to me a lot and helped me understand many things. He told me that I am not the first or the last who is going through this. I had fear for my family as well because they were asking me how I was and what happened. Only my 3 daughters know about it. I did not want to know anything about anyone, because I had fear of restarting my life. I had a Spanish boyfriend and he did not know either.”*

In the same vein, Participant 5 described experiencing distress due to the process of assuming the diagnosis and sharing it with the family, and also dealing with the treatment side effects.

Participant 5: *“( …) They told me that I was HIV-positive and you can imagine what I was thinking, oh my God, my little children, my husband. They told me do not worry, that you can last for 10 years. Imagine it was 2003, I had 7- or 8-year-old kids and another who was 12 or 13 years old. So little, and imagine they tell you this, that you can last 10 years, my head went crazy. In the end I started the treatment, but it gave me an incredible allergy. I could not wear clothes because everything was itching, another [medication] made me vomit, and I could not walk, but I was looking in the mirror and every time I was seeing myself marked by another treatment. Because all treatments were hurting me I stopped taking them and I spent one and a half years without any treatment before I came to Spain. The second treatment that they gave me here was effective ( …) My friends did not know because there [referring to her country of origin] you could not say this. Sincerely I did not have any support.”*

Similarly, other participants, such as Participant 7, expressed difficulties due to lack of treatment in their countries of origin, and had the following reaction after being diagnosed:

*“I will die. I thought that I will die.”*

Another participant expressed that it was necessary to be positive; however, it was hard to initially adjust to the knowledge of being HIV-positive.

Participant10: *“If you are positive, it will give you a lot of fear; you are scared to enter this world, because it is not easy. You go to bed thinking that you have the disease and you wake up knowing you have the disease and it is hard. In the beginning it is hard to overcome this thinking, it is hard to overcome, but when you accept it then you know that you will live with this until the end of your days.”*

A few participants did not express having strong emotional distress due to being diagnosed with HIV. They took care of themselves (received treatment, lived a healthy lifestyle); however, they nonetheless avoided sharing information with others.

Participant 1: *“When I found out about it, I knew I had to live a bit differently, and I did not get upset. So, I was taking care of myself. Especially when I was not taking any medication, so I ate well and slept well. Because I did not have a medication, I was limiting my life a bit. I do not feel like I have HIV. I go on as if nothing happened to me.”*

Few participants expressed no or limited emotional distress while dealing with their diagnoses or life as HIV-positive persons, contrary to the majority of immigrants who were interviewed. For example, Participant 3 shared the following:

Participant 3: *“( …) I got it and that is it, it is something that you do not choose ( …) you have to accept it. I think I accepted it quite well. However, it was still a process of adaptation ( …) I know that I have it, but it is controlled ( …) sometimes I do not believe that I have it. I know that it is there and I take care of myself, I take my medication ( …)”.*

Another participant explained that, although it was hard to adapt to the idea of being HIV-positive, the support of her partner made it manageable for her.

Participant 6: *“For me it was hard, it is a hard situation. Knowing that you are limited in many things ( …) but I was lucky to have a partner, we were together, we were in the same situation ( …) I cannot imagine people who are alone, people who do not have support of a partner or family members or of a friend or similar, because friends discriminate too.”*

#### Theme 4: perceived or experienced discrimination due to being HIV-positive in Spain

The majority of participants experienced discrimination or expressed fear of possible discrimination due to their HIV status. For example, Participant 12 said the following:

Participant 12: *“We went to a pool in a village and the owner told his subordinates not to let us in the pool because we will contaminate everyone with AIDS. In the hospital as well. I was in the observation room and I asked someone for water. He was slow to bring it and I heard them talking. I heard one worker telling another that he should be careful with me because I have HIV. I got angry and I told them, I will not contaminate your fellow worker if he brings me a glass of water.”*

Participant 11 also noted how she was treated differently due to prejudice or lack of knowledge about HIV and how it could be transmitted.

Participant 11: *“I lived with my niece who was living with her aunt. This woman knew of my health condition but her children did not. Once I was cooking a stew and I took a spoon to try it and she [referring to the aunt] thought I was planning to put it directly to my mouth. She told me “put it in your hand.” I told her, “I was planning to do that.” I saw where this was going. She was asking, “How are you?” “You have to be careful with these things.” Of course, I was very careful, including after I showered. I leave the bathroom as if no one has entered it.”*

Some participants described having fear of discrimination in different situations in their lives in Spain.

Participant 10: *“It is a country with people of different ages, and you cannot compare a person who is 80 or 70 years old with one who is 20 years old. Older people bring a lot of stigma and taboos to many situations. It does not matter how much you say that Spain is free and is diverse and there are a lot of liberties, the society here is still trying to accept things.”*

Participant 10 also talked about perceived fear of being denied employment or being fired because of his HIV status.

Participant 10: *“One also has fear in an environment of employment when you think” “They will do a test for HIV and it will come out positive, and they will fire me, they will not want me, they will reject me.”*

Similarly, Participant 2 described her actual experience when trying to get a job.

Participant 2: *“Recently, in some jobs they no longer discriminate because of age and HIV status, because in some other jobs they continue to reject you for other reasons, but in reality, because they do analysis and HIV status comes out positive. Because they want to be sure that people will not miss work, contracts are short, they want to get rid of a lot of problems. They think that people who have HIV will be constantly in the hospital.”*

On the other hand, Participant 8 described a violation of her right to doctor-patient privacy because she had HIV.

Participant 8: *“My partner did not know it [referring to her HIV status], and when I told my doctor, I felt liberated. This doctor made me feel safe, but when I left the room and my partner entered, he told him “How is it going with your partner, so many years as a HIV patient?” When he came out of the room, he gave me a look. I wanted to report him [referring to the doctor] because it should not be like this, because supposedly I am signing a document that says that whatever I tell my doctor is confidential.”*

A few participants expressed limited or no experience of discrimination or perceived discrimination due to being HIV-positive, however their responses were nonetheless telling of the broader social conditions around HIV. For example, Participant 1 shared the following:

Participant 1: *“Because I never shared and I never talked about it, I never had this experience.”*

### Possible influence of other variables on positive experience

The study also looked at the possible influence of the documentation status of the participants and/or of the type of guidance received on positive or negative experiences of applying for a public health insurance card. All participants who arrived in Spain with legal documents (5 of 12) or as an asylum seeker (2 of 12) had a positive experience when applying for a public health insurance card. Four out of these 7 participants received guidance from an NGO through the process, 2 were assisted by friends, and 1 was aided by a hospital staff member.

Of the 5 participants who arrived in Spain undocumented, 3 had negative or somewhat negative experiences while applying for their public health insurance cards. Two undocumented participants had a positive experience. Of these 2 latter participants, 1 was guided through the process by an NGO and another was guided by a friend who initially was also assisted by an NGO. Of the 3 participants who had negative or somewhat negative experiences, 2 had minimal guidance from a friend and resolved the obstacles by themselves. The third undocumented immigrant had to access emergency care due to an accident for which she was billed. After receiving the bill, she was advised by a family member not to pay the bill. This participant already had legal immigrant status in Spain when she needed healthcare services for the first time.

## Discussion

The number of immigrants in Spain has increased greatly over the past decade. In 1998, immigrants were only 1.6% of the total population; by 2019, the percentage had increased to 10.7% [11]. It is commonly observed that immigrants arrive healthy in the country (healthy immigrant effect) [41, 42]; however, with the passage of time, their health worsens due to various socioeconomic factors and to the risk of being excluded from free public health services [43, 44]. Few studies have examined the process of access to healthcare services among HIV-positive immigrants in Spain [45]. To our knowledge, this is one of the first studies to examine the experiences of this vulnerable population in Spain, especially after the passage of 2012 RDL and RD. This study adds new knowledge to the public health literature, highlighting the personal and systemic struggles faced by HIV-positive immigrants in Spain, in both health care and other arenas.

We found that all participants who encountered barriers while applying for a public health insurance card mentioned not having proof of registration in an AC (with or without the required minimum time period of residence) as an obstacle (Theme 1). Participants who had a somewhat positive experience of applying for a public health insurance card were guided by a social worker, a friend, or family member or an NGO/association (Theme 2). Participants who had somewhat negative experiences while applying for a public health insurance card reported less guidance from another person or organization. The majority of the participants did not appear to have knowledge of the 2012 health reform in Spain and thus did not express its effect on their lives.

Results are consistent with other studies that identified difficulties individuals had meeting legal requirements to use free healthcare services, including acquiring a public health insurance card, which were barriers to access to care in Spain. Being an undocumented immigrant has also been identified as a risk factor for facing more barriers while accessing healthcare services [46, 47]. The 2012 RDL and RD abolished previously practiced universal healthcare access in Spain [48], and the requirement of being registered in an AC, together with the need of an identification document, were barriers stemming from the new law. Therefore, regardless of whether participants were aware of the 2012 RDL and RD, the commonly cited barrier to accessing a public health insurance card was the direct effect of 2012 RDL and RD.

This study highlights the importance of the existence of NGOs and social networks in facilitating HIV-positive immigrants’ access to free healthcare services. Even when individuals are not directly engaged with an NGO, it can still be the source of informational or instrumental support for family and friends who try to help. NGOs appear to be an important safety net system in Spain and appear to buffer against restrictions that Spanish laws imposed on healthcare access among undocumented immigrants. We have found previously that regional implementation of the national laws varied greatly, with some ACs eventually granting access to undocumented immigrants who were HIV-positive (Gogishvili et al., unpublished data), but navigating these provisions was often difficult for individuals. Previous studies have also shown that third-party guidance is a positive facilitator for entry into the healthcare system in Spain [47, 49]. Various NGOs and medical communities in Spain have been actively involved in advocating for the rights of immigrants to health care, especially after the implementation of 2012 RDL and RD [48]. Some of these organizations have also been providing administrative support or healthcare assistance to immigrants who were initially denied care [49]. Furthermore, having a robust civil society may be crucial for mobilizing advocacy and policy action in the face of political ideology that is not public health-friendly in many ACs; political ideology can often influence how each AC implements national laws on healthcare access [50].

Experiences (or fear) of discrimination, stigma, and intolerance have been previously reported by HIV-positive immigrants in Spain and other European countries [39, 47], as has the burden and struggle of their health status [39]. In addition, lack of family support or fear of sharing their health status with loved ones (due to stigma and prejudice) by some participants of this pilot study also demonstrates multiple dimensions of struggle and distress HIV-positive immigrants go through, on their own, beyond commonly described discrimination patterns experienced outside their close circles. More in-depth qualitative research is warranted to explore these multiple dimensions of experiences. This also highlights the importance of disseminating information on available support services (hospitals, health centers, pharmacies, other community organizations) for all HIV-positive immigrants and on their right to health, especially because many immigrants come from countries with stigma and intolerance regarding HIV.

Discrimination due to misconceptions about HIV transmission by the general population is another dimension that needs addressing in Spain. As described by some participants in theme 4, they have experienced discrimination in Spain because of ignorance about HIV and its routes of transmission. Previous study has demonstrated that while misconception about HIV transmission has slightly improved over time in Spain, some unwarranted beliefs such as the possibility of transmission through social contact persist [51]. Such misconceptions, and associated prejudices, can exacerbate the discrimination experienced by HIV-positive immigrants [52]. The need for comprehensive systems intervention to address the multifaceted challenges faced by HIV-positive immigrants in Spain is evident and is reinforced by findings from this study.

We recognize some limitations of our study. First, our sampling relied on staff from the collaborating NGO who might not have reached out widely to individuals who did not speak Spanish or who were from sub-Saharan Africa. Thus, our findings are only valid in the specific group of participants based on their background characteristics. Another limitation is the possible impact of the face-to-face interview process on underreporting of participants’ experiences as HIV-positive persons living in Spain. However, the interviewer came from a social work background and created a safe and motivating environment for the interviewees in which to freely tell their stories. Finally, although questions for the interviews were designed specifically for this study, it is possible that a different set of questions could have given different results. For example, due to the healthcare context of the interview, participants might have leaned toward discussing discrimination experienced in relation to their HIV rather than immigrant status. Nevertheless, the interviews were semi-structured, which allowed interviewees to organically free associate and deviate from the questions as needed. This allowed exploration of participants’ lives as HIV-positive immigrants in Spain that was not initially included in the semi-structured interview guide.

## Conclusions

This study is a contribution to the limited literature describing the personal journeys of HIV-positive immigrants and their experiences accessing healthcare services in Spain, especially after the passage of 2012 RDL and RD. Immigrants represent a growing population in Spain, and all of Europe, and greater public health attention to the needs of such vulnerable populations is urgently needed. The results of this study demonstrate the important role of NGOs in helping HIV-positive immigrants navigate the system. The study also highlighted discrimination experienced by HIV-positive immigrants in Spain due to misconceptions about HIV transmission and perceived stigma about the infection that followed them from the time of diagnosis. The results clearly demonstrate multifaceted challenges faced by HIV-positive immigrants at both personal and policy levels. Future follow-up research is needed to design and implement effective models of improving the care and health of HIV-positive immigrants in Spain and throughout Europe.

## Figures and Tables

**Fig. 1:**
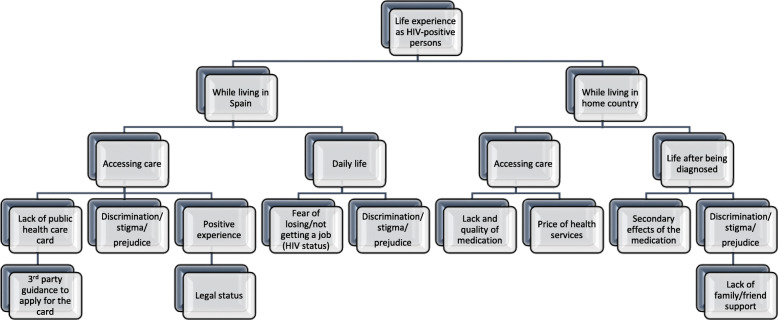
Coding Tree

## Data Availability

The datasets generated and/or analyzed during the current study are not publicly available due to the small sample size and personal and confidential nature of qualitative information.

## Appendix 1

### Semi-structured Interview Guide Script

A. **Personal information**

**The aim of this guide is to describe the life narrative of the immigrant.**

1. **Please tell me about you:**
  - Your full name
  - Age
  - Sex
  - Country of birth
  - Family situation (marital status, if all family members are here with you in Spain)
  - Education
2. **Please tell me about your current situation:**
  - Your current professional position/ economic situation
  - Number of years in current position (if applicable)
  - Legal or administrative status or issues in Spain
  - Language knowledge
3. **Tell me about your experience of immigration to Spain:**
  - Country of origin
  - Reason of immigration
  - Immigrated alone or with other family members, friends
  - In which autonomous community and city or village do you reside?
  - Approximately since when have you permanently resided in Spain: MM/YYYY
  - Issues or positive experiences in immigrating and adapting to living in Spain

B. **Information about HIV**

**The aim of this part is to understand HIV disease awareness of the immigrant, to collect information on his/her sexual life, and to understand what his/her initial experiences were during and after getting diagnosed as HIV-positive.**

1. **What is your knowledge about HIV?**
  - Transmission
  - Prevention
  - Treatment
2. **Can you tell me about your sexual partners?**
  - Sexual orientation
  - Number of partners (if applicable)
3. **What is your experience of living with HIV?**
  - Why did you get tested for HIV?
  - Did you know where to go to have an HIV test done?
  - Approximately when you were diagnosed HIV-positive and where: MM/YYYY
  - Did someone explain to you what it means to have HIV and how to manage it, or what were the next steps? Was it clear for you which services you would need to access after you were diagnosed HIV-positive and where to go?
  - What is your perception of HIV infection
  - Do your family and friends know you are HIV-positive? Do they support you in managing the infection?
  - Did your life change after you were diagnosed as HIV-positive? How? Did it affect your relationships with family members, friends or coworkers?
  - What are your experiences with discrimination due to your HIV-positive status?
  - What communication do you have with or knowledge of other people living with HIV-positive status in Spain or back home?

C. **Usage and access to necessary services**

**The aim of this part is to determine which services the immigrant is utilizing and what barriers he/she is encountering (or experienced in past) while accessing necessary testing or healthcare.**

1. **Which healthcare system are you utilizing at the moment?**
  - Public healthcare
  - Private insurance
  - None of the above
  If, none of the above where do you go or what do you do when you need medical care?
2. **Where you tested for HIV in Spain?**
  If yes:
  - Did you know where to go?
  - Describe your experiences (accessing a testing facility, how were you treated, how was information delivered to you)
  - Do you know if you were late-to-diagnose? Late to treat?
3. **Are you currently in treatment for HIV or a related health condition?**
  If yes:
  - What treatment are you currently on?
  - Why are you using the service provider that you are using?
  - Describe your experiences (accessing services, how were you treated, how was information delivered to you)
4. **Are you paying for the services provided for HIV treatment or other related health conditions?**
  If yes:
  What is the percentage of the fee you are paying?
  Was there ever a case when you could not pay? What happened?

D. **Information and effect of the 2012 healthcare reform**

**The aim of this part is to determine how much information the immigrant was provided or understood about limitations imposed by the 2012 healthcare reform and how and/or if it changed their experience of accessing necessary healthcare services.**

1. **Were you in Spain in 2012?**
2. **Were you getting HIV-related healthcare in Spain in 2010? Or in 2018?**
3. **Can you tell me which healthcare services you think you are entitled to access free of charge and for which services you have to pay or co-pay?**
  - Emergency care
  - Blood tests, radiology, genecology, urology, or other health services necessary upon need (not including dentist, and/or any services that are more esthetic procedures than health related).
  - HIV testing, pre-exposure prophylaxis treatment, antiretroviral treatment, follow-up visits, health guidance related to HIV infection.
4. **Did you ever had difficulties in accessing healthcare services?**
  If yes:
  - Where (Spain, your home country)?
  - When (approximately what year)?
  - Why (inability to pay, denied by healthcare professional, not sure where to seek required care)?
5. **Where you ever been denied right to healthcare services?**
  If yes:
  - Where (Spain, your home country)?
  - When (approximately what year)?
  - Why (inability to pay, denied by healthcare professional, not sure where to seek required care)?
  - What was the explanation given to you?
6. **Are you aware of the limitations imposed by the 2012 health reform on immigrants?**

7. **Are you aware of changes made to the 2012 healthcare reform in Valencia?**

## Abbreviations

HIV: Human Immunodeficiency Virus
RDL: Royal Decree Law
RD: Royal Decree
NGO: Nongovernmental Organization
EU: European Union
AVACOS-H: Asociación Valenciana de VIH, SIDA y Hepatitis (Valencian Association dedicated to HIV AIDS, and Hepatitis)
AIDS: Acquired Immunodeficiency Syndrome
AC: Autonomous Community
